# The Earliest Writer on Carbolic Acid

**Published:** 1869-11

**Authors:** 


The Earliest Writer on Carbolic Acid.—We are indebted to Dr. Lemaire, of Paris, for the earliest publications on carbolic acid. In 1861, three essays on its uses were published by him, and in 1863 he wrote an interesting monograph on its uses in surgery and medicine.

